# Prolonged prone position ventilation for SARS-CoV-2 patients is feasible and effective

**DOI:** 10.1186/s13054-020-02956-w

**Published:** 2020-05-15

**Authors:** Andrea Carsetti, Agnese Damia Paciarini, Benedetto Marini, Simona Pantanetti, Erica Adrario, Abele Donati

**Affiliations:** 1 Department of Biomedical Sciences and Public Health, Università Politecnica delle Marche, Ancona, Italy; 2 Anesthesia and Intensive Care Unit, Azienda Ospedaliero Universitaria Ospedali Riuniti, Ancona, Italy

**Keywords:** SARS-CoV-2, Prone position ventilation

Recently, novel coronavirus 2019 (nCOV-19) is spreading all around the world causing severe acute respiratory syndrome (SARS-CoV-2) requiring mechanical ventilation in about 5% of infected people [1, 2]. Prone position ventilation is an established method to improve oxygenation in severe acute respiratory distress syndrome (ARDS), and its application was able to reduce mortality rate [3]. Although the severity of critically ill patients with SARS-CoV-2 may require pronation [4], the huge number of patients requiring intensive care unit (ICU) admission may create management problems due to the limited number of healthcare workers compared to the number of patients. Often, sustained oxygenation improvement can only be achieved after several cycles of pronation, with a work overload for healthcare staff. To face these problems, we implemented a pronation protocol that allows to extend the time for the prone position beyond 16 h, aiming to reduce the number of pronation cycles per patient. Thus, the aim of this report was to assess the feasibility and efficacy of prone position ventilation beyond the usual 16 h.

We retrospectively collected data from 10 critically ill patients intubated and mechanically ventilated for SARS-CoV-2. Six patients underwent both standard and prolonged pronation, the latter after one standard cycle failure; 3 patients underwent prolonged pronation only and 1 patient just to the standard one. We recorded PaO_2_/FiO_2_ values before pronation (T0), during pronation (T1), and in the supine position after the pronation cycle (T2). Friedman’s test has been used for comparisons, considering a *p* value < 0.05 as significant.

All patients were male, with a median age of 58 years (IQR 50; 64). Six patients (54.4%) were obese. All standard pronation cycles lasted for 16 h whereas the median duration of prolonged pronation cycles was 36 h (IQR 33.5–39). Ventilatory parameters before the first pronation trial are listed in Table 1. Oxygenation significantly improved during ventilation in prone position (Fig. 1). Interestingly, PaO_2_/FiO_2_ recorded in the supine position after a prolonged pronation trial was significantly higher than PaO_2_/FiO_2_ measured before pronation (*p* = 0.034). On the other hand, the gain in oxygenation was not maintained after the standard pronation cycle (*p* = 0.423). Static compliance of the respiratory system did not change significantly following prone position ventilation (*p* > 0.05). Application of prolonged prone position did not expose patients to an increased incidence of skin pression lesions, and other complications were not reported.

**Table 1:** Patients’ baseline characteristics

FiO_2_	0.7 (0.18)
PEEP (cmH_2_O)	14 (1.49)
Pplat (cmH_2_O)	24 (1.94)
∆*P* (cmH_2_O)	9.5 (2.87)
Cstat (ml/cmH_2_O)	49 (9.24)
PaO_2_/FiO_2_ (mmHg)	119 (33.65)

Data reported as mean (standard deviation)

*Cstat* static compliance of the respiratory system, *∆P* driving pressure, *FiO*_*2*_ fraction of inspired oxygen, *PaO*_*2*_ arterial partial pressure of oxygen, *PEEP* positive end-expiratory pressure, *Pplat* plateau pressure

**Fig. 1:**
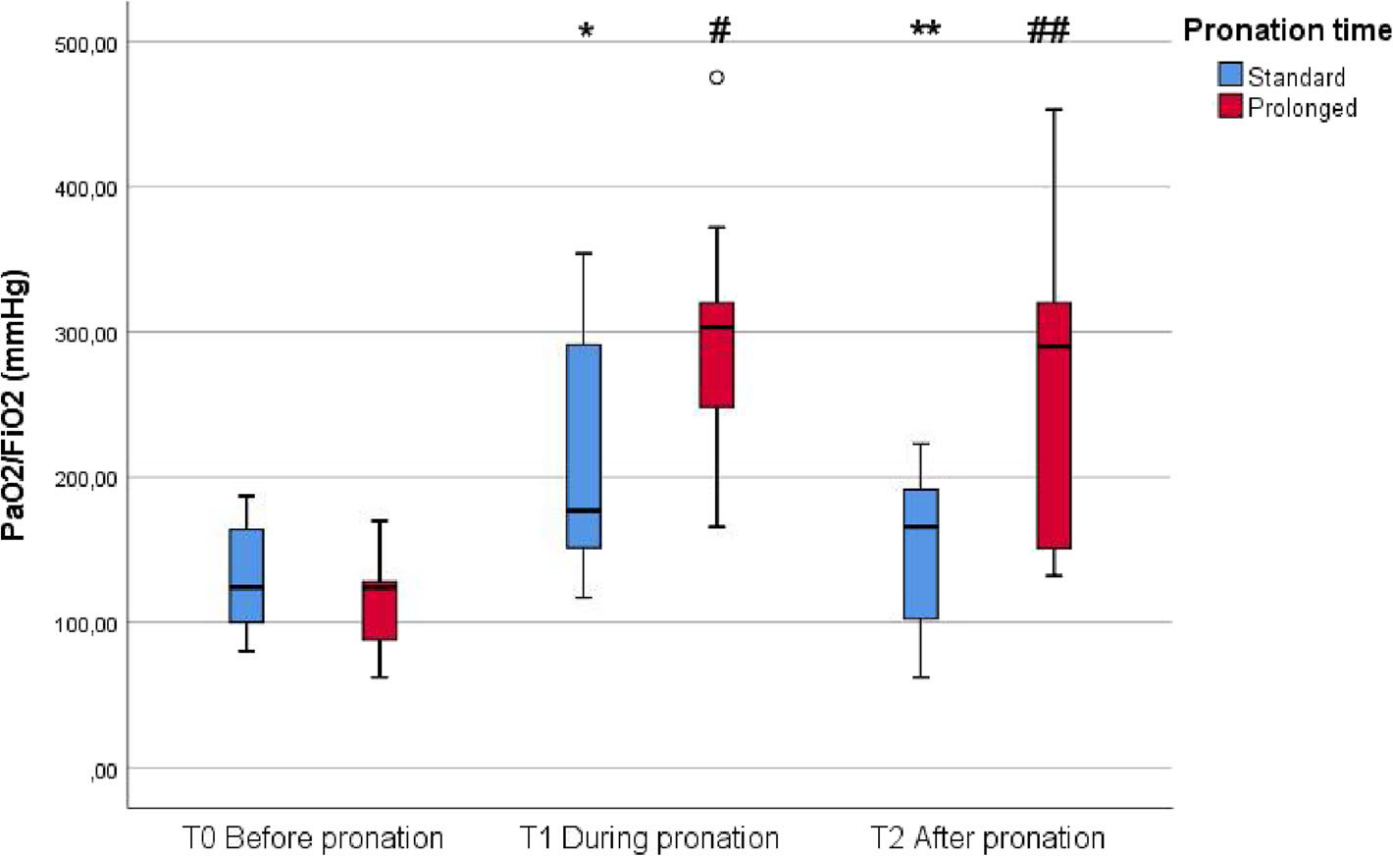
PaO_2_/FiO_2_ comparison between standard and prolonged prone position ventilation. *Standard pronation: T1 vs. T0, *p* = 0.01; **standard pronation: T2 vs. T1, *p* = 0.016; ^#^prolonged pronation: T1 vs. T0, *p* < 0.001; ^##^prolonged pronation: T2 vs. T0, *p* = 0.034

Our report showed that prone position beyond 16 h may probably be safely performed in patients with SARS-CoV-2 and severe hypoxemia not responsive to conventional mechanical ventilation. This approach might have several potential advantages. First, oxygenation improvement might be higher during prolonged pronation than during standard pronation, and the gain might be more sustained over time. Second, in the condition of work overload for healthcare assistants, this strategy might reduce the number of pronation cycles needed for a single patient. Finally, no adverse events have been observed following this approach. However, a well-trained healthcare team is mandatory to perform the procedure, to rapidly face potential complications, and to guarantee appropriate patient preparation to reduce the risk of bedsore lesions. The team experience may be a potential problem during the pandemic because the recruitment of staff without a critical care background may be needed in ICUs to cope with personnel shortage. Obviously, these data must be interpreted with caution and need to be confirmed because of the small number of patients considered and the retrospective design of the study.

In conclusion, we showed that prolonged prone position up to 36 h is feasible, safe, and may offer potential clinical and organizational advantages.

## Data Availability

The datasets used and/or analyzed during the current study are available from the corresponding author on reasonable request.

## Abbreviations

∆*P*: Driving pressure
ARDS: Acute respiratory distress syndrome
Cstat: Static compliance of the respiratory system
FiO_2_: Fraction of inspired oxygen
ICU: Intensive care unit
IQR: Interquartile range
nCOV-19: Novel coronavirus 2019
PaO_2_: Arterial partial pressure of oxygen
PEEP: Positive end-expiratory pressure
Pplat: Plateau pressure
SARS-CoV-2: Severe acute respiratory syndrome
